# Thyroid Rosai-Dorfman disease with infiltration of IgG4-bearing plasma cells associated with multiple small pulmonary cysts

**DOI:** 10.1186/s12890-019-0847-1

**Published:** 2019-05-03

**Authors:** Pietro Gianella, Nicolas Dulguerov, Grégoire Arnoux, Marc Pusztaszeri, Jörg D. Seebach

**Affiliations:** 1 Division of Pulmonary Diseases, Geneva University Hospitals, 4 Rue Gabrielle-Perret-Gentil, 1211, 14 Geneva, Switzerland; 2 Division of Head and Neck Surgery, Geneva University Hospitals, Geneva, Switzerland; 3 Department of Clinical Pathology, Geneva University Hospitals, Geneva, Switzerland; 4 Division of Immunology and Allergy, Geneva University Hospitals, Geneva, Switzerland

**Keywords:** Rosai-Dorfman disease, Non-Langerhans cell histiocytosis, Lungs cystic lesions

## Abstract

**Background:**

Rosai-Dorfman disease (RDD) is a rare histiocytosis which involves principally lymph nodes. Thyroid involvement in RDD is a very rare situation, and lung involvement is even rarer.

**Case presentation:**

We report the case of a 46-year-old woman presenting a painless mass in the right side of the neck and subacute dyspnoea. Computerised tomography (CT) scans of the neck and thorax showed a large thyroid mass causing tracheal stenosis and multiple cystic lesions in both lungs. Subtotal thyroidectomy with a tracheal segment resection and histological analysis confirmed the diagnosis of nodal and extranodal (thyroid, tracheal and probably lung) Rosai-Dorfman disease (RDD) with the presence of increased numbers of IgG4-bearing plasma cells. Clinical, functional and radiological follow up 4 years after surgery without medical treatment did not show any disease progression.

**Conclusions:**

This case report indicates a benign course of nodal RDD with thyroid and tracheal infiltration following surgical resection, association of typical histological signs of RDD (emperipolesis) with IgG4-related disease features, and that lung cysts might be a manifestation of RDD.

## Background

According to the recent classification published by Emile and colleagues Rosai-Dorfman disease (RDD), also known as sinus histiocytosis with massive lymphadenopathy, is a rare non-Langerhans cell histiocytosis which involves principally lymph nodes [1]. Diagnosis depends on biopsies with immunostaining demonstrating infiltration with histiocytes positive for CD68+/S100+, negative for CD1a and CD207 excluding pulmonary Langerhans cell histiocytosis (PLCH); and the presence of intact lymphocytes within the histiocyte cytoplasm (known as emperipolesis). Although emperipolesis is not pathognomonic and can be seen in other conditions, most notably Erdheim-Chester disease and hemophagocytic lymphohistiocytosis, it is highly suggestive for RDD. Extranodal involvement occurs in up to 43% of cases with skin, central nervous system, and salivary glands as the most frequently reported sites. Only a few hundred cases of RDD are altogether reported in the literature [2–4]. Thyroid and respiratory tract involvement has been described in less than 3% [3, 5, 6], with only one case of concomitant thyroid and lung involvement [7]. Besides mediastinal lymphadenopathy, tracheal and bronchial involvement, nodular masses, bronchiectasis, pleural effusion, and interstitial infiltrates have been reported. In the majority of cases, RDD has a benign course and treatment is not necessary. Therapy is required for patients with extranodal RDD having vital organ involvement or those with nodal disease causing life-threatening complications. Complete remission can often be achieved after surgical excision. Systemic corticosteroids are the most effective medical treatment in case of systemic symptoms or symptomatic lymph nodes enlargement [3, 4].

## Case presentation

A 46-year-old never smoker Malagasy woman was referred by her family doctor to the emergency department due to dyspnoea with inspiratory stridor and inspiratory-expiratory wheezing with insidious onset over a 3-month period. The patient was treated for supposed asthma since 2 weeks without improvement. She had no fever, weight loss or night sweats. Physical examination revealed a heart rate of 96/min, a respiratory rate of 19/min, and an oxygen saturation of 89% on room air with normal chest auscultation. A non-tender mass was detected on the right side of her neck.

**Fig. 1:**
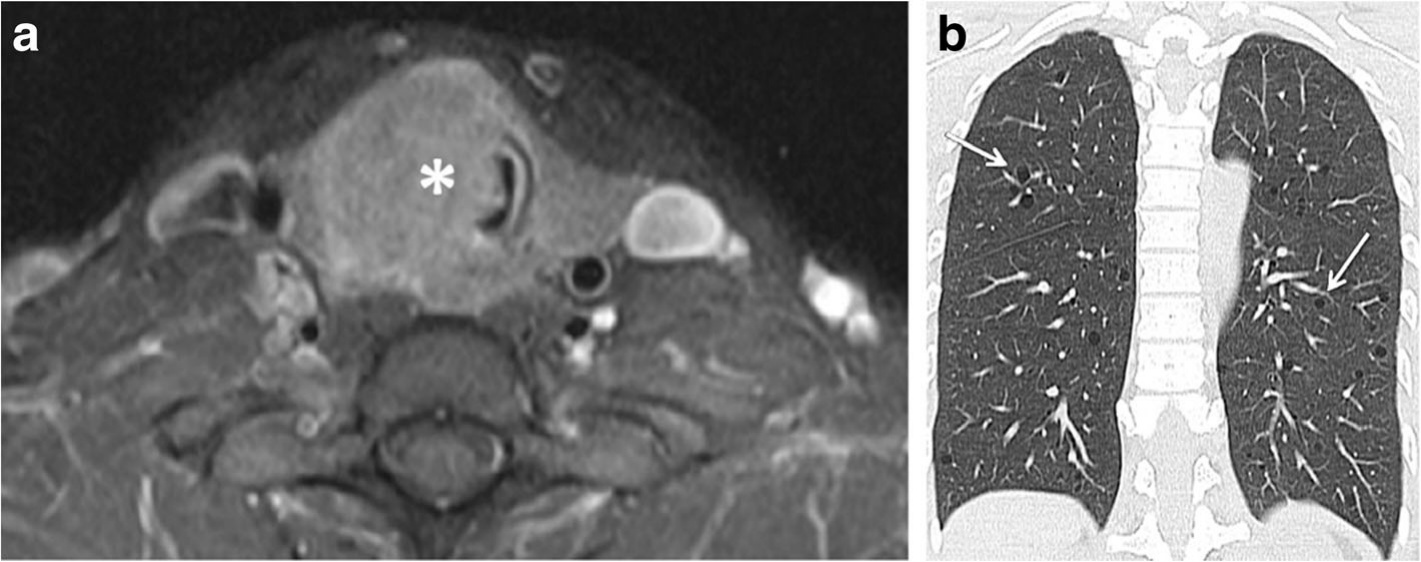
Neck CT scan (axial reconstruction) showing a large right sided thyroid mass (asterisk) which caused marked tracheal deviation and stenosis (**a**). Thorax CT scan (coronal reconstruction) showing multiple cystic lesions with thin wall in both lungs (arrows) (**b**)

CT scans of the neck and thorax showed a large thyroid mass causing tracheal stenosis (Fig. 1a), and multiple cystic lesions with thin walls in both lungs (Fig. 1b). Cysts had a diffuse localisation, including the costophrenic recesses. Neither pulmonary nodules nor ground glass opacities were observed. Abdominal CT scan did not show any sign of renal angiomyolipoma.

Blood arterial gases showed mild hypoxemia (PaO2 82 mmHg). Laboratory investigations, including renal function, liver function tests, C-reactive protein, thyroid function tests, complete blood cell counts, and serum IgG4 levels were all within normal limits. HIV and immunological tests (anti-Ro/SSA and anti-La/SSB antibodies, rheumatoid factor and thyroid-stimulating hormone (TSH) receptor antibody) were negative. Plasma levels of vascular endothelial growth factor-D (VEGF-D) were low (347 pg/mL; *normal range 0–450 pg/mL*). Electrocardiogram (ECG) tracing and complete pulmonary function testing were normal (forced expiratory volume in 1 s - FEV1 94%, total lung capacity – TLC of 95% of predicted and normal carbon monoxide diffusing capacity - DLCO).

**Fig. 2:**
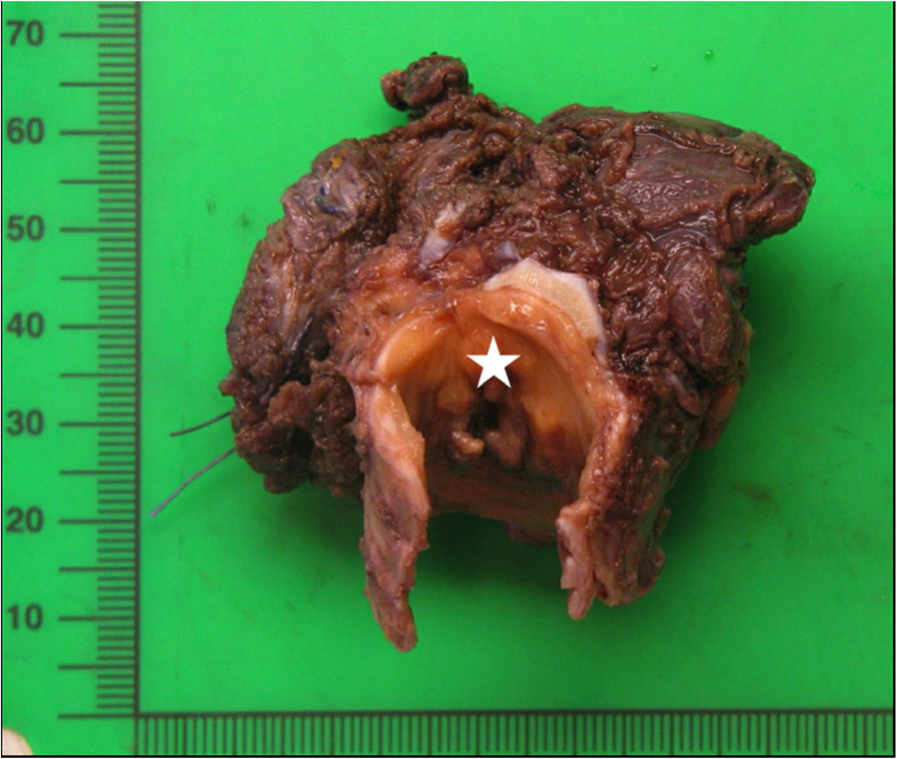
Surgical specimen showing a poorly delimited infiltrative whitish thyroid mass with extension to the adjacent tracheal structure. Star indicates the luminal surface of the trachea

**Fig. 3:**
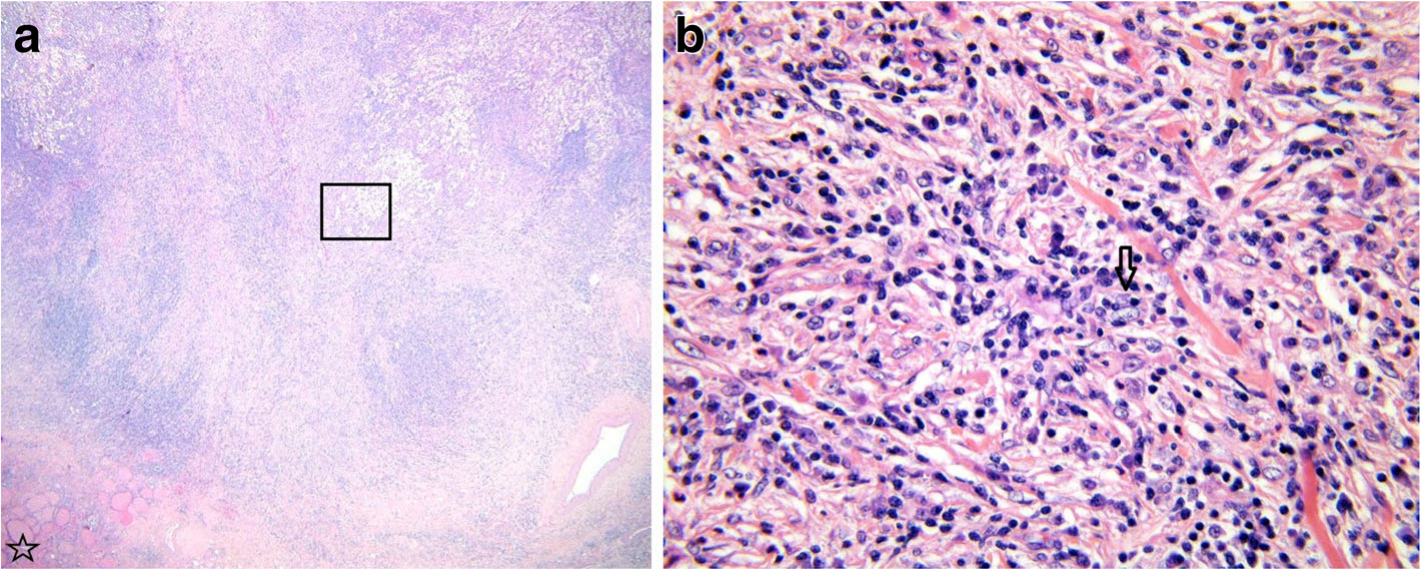
**a** Overview of one representative fragment showing a dense fibro-inflammatory process involving the thyroid and the tracheal wall (boxed area). The star indicates residual thyroid tissue. **b** Detail view of the boxed area in (A) showing predominant histiocytic infiltrates along with numerous emperipolesis figures, i.e. histiocytes engulfing neutrophil granulocytes and lymphocytes (arrow head). This combination is a prominent feature of Rosai-Dorfman disease. H&E staining; original magnification: A × 20; B × 400

**Fig. 4:**
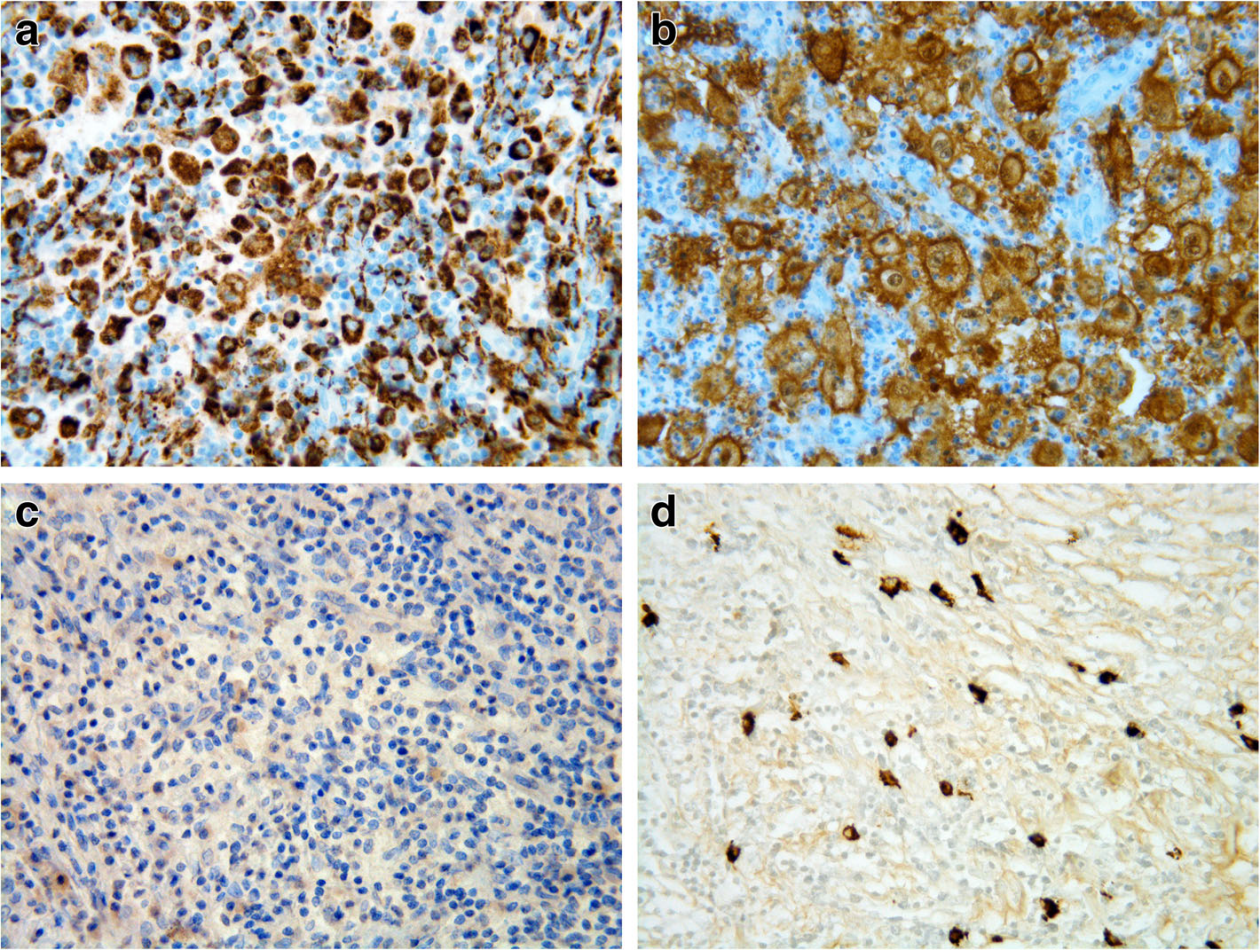
Detailed view of the immunostaining results showing histiocytes strongly positive for CD68 (**a**) and S100 (**b**), negative staining for CD1a (**c**), and increased numbers of IgG4-bearing plasma cells (**d**). Original magnification A-D × 400

Surgical removal of the thyroid mass with subtotal thyroidectomy, tracheal segment (3.5 cm) resection and multiple adenectomies were performed (Fig. 2). Microscopic study revealed extensive infiltration of all lymph nodes and the thyroid with partial replacement of follicular parenchyma by a fibro-inflammatory process of mixed cellularity, rich in histiocytes (Fig. 3a). The latter exhibited epithelioid to xanthomatous morphology and formed ill-defined clusters or confluent sheets without granulomatous pattern. An important fraction of the histiocytic population showed emperipolesis of neutrophil granulocytes and lymphocytes (Fig. 3b). Immunohistochemistry revealed co-expression of the macrophage-related epitope CD68 along with S100 protein, in the absence of CD1a, thereby identifying the histiocytic elements as of non-Langerhans lineage (Fig.4a, 4b and 4c). Remarkably, storiforme fibrosis with signs of vasculitis was also observed and immuno-phenotyping of the infiltrate revealed a substantial participation of IgG4-bearing plasma cells. While their absolute density reached up to 50 per high power field, the IgG4:IgG ratio did not exceed 20% (Fig.4d). Histological examination of the tissue biopsies for the identification of infectious organisms using several stainings for pathogens were negative. Based on these findings, a histological diagnosis of RDD was made.

Following surgery dyspnoea disappeared indicating that it was related to tracheal compression. The patient was asymptomatic in the absence of any specific treatment; clinical, functional lung testing and radiological follow up 4 years after surgery did not reveal any disease activity or progression underlining the excellent prognosis of the disease after resection.

## Discussion and conclusions

Our patient presents the very uncommon association of thyroid, tracheal and nodal RDD with multiple small lung cysts. Only few cases of lung cysts associated with RDD are described in the literature. In fact, a recent Mayo case series of 9 patients with intrathoracic manifestations of RDD mainly found mediastinal lymph node involvement. However, there were two patients (#4 and #9) with lung cysts and bronchiectasis [6]. Moreover, cystic lung disease has developed over time in a patient presenting with consolidative nodules, pleural thickening, septal lines, and mediastinal lymphadenopathy. The diagnosis of pulmonary RDD was established by open-lung biopsy [8]. Another patient with cutaneous RDD showed bilateral pulmonary cysts and numerous ground glass nodules with characteristic upper lobe predominance. There was no intrathoracic lymphadenopathy or pleural effusion mimicking PLCH. However, the biopsy of the concomitant skin lesion confirmed the histological diagnosis of RDD [9]. Thus, cyst-formation is possible in pulmonary RDD, it is however very rare and likely to be accompanied by other pulmonary radiographic findings, suggestive of interstitial lung disease. The main differential diagnosis of pulmonary cysts included PLCH, lymphocytic interstitial pneumonia (LIP), or lymphangioleiomyomatosis (LAM) [10, 11]. In fact, histiocytosis (e.g. PLCH and Erdheim-Chester disease) frequently cause cystic lung disease with a mid to upper zone predominance [12]. Nevertheless, the absence of tobacco consumption, the diffuse cysts localisation and the absence of peribronchiolar nodular opacities all argued against PLCH. Moreover, the patient did not present any of the typical features of Erdheim-Chester disease. The clinical presentation as well as the absence of pulmonary nodules and ground glass opacity was atypical for LIP. Furthermore, human immunodeficiency virus (HIV) and immunological tests (anti-Ro/SSA and anti-La/SSB antibodies, rheumatoid factor and TRAb) were negative. Genetic testing for Birt-Hogg-Dube syndrome (BHDS) was not performed since there was no family history, no extrapulmonary manifestations, such as skin lesions or renal cancer, and the distribution of the cysts was not suggestive. Lung cysts in BHDS are multiple, thin walled, typically seen in the peripheral zones at lung bases and along the mediastinum with a disproportionate number of paramediastinal elliptical (floppy) cysts, and can abut or encase the proximal portion of the lower pulmonary veins [11]. In contrast, the multiple small and thin-walled lung cysts in the presented case were higher in number and diffusely distributed throughout the lung parenchyma, more ressembling the distribution in LAM. The radiologic appearance of the cysts, age and sex of the patient were indeed compatible with LAM. The VEGF-D plasma level was low (347 pg/mL) neither confirming nor excluding LAM since VEGF has a good positive predictive value if the value is greater than 600 pg/mL but a poor negative predictive value below this threshold [13]. In addition, abdominal CT scanning did not show any sign of renal angiomyolipoma which is associated to LAM in 50% of the cases. Taken together, without lung biopsy the observed cystic lung lesions cannot with certainty be attributed to RDD, since a concomitant diagnosis of LAM or BHDS was not completely ruled out. Although two rare diseases may coexist in the same patient, it is preferable to retain a single etiology and unifying diagnosis. Therefore, in analogy to other histiocytosis known to cause lung cysts the pulmonary lesions in our case are probably related to RDD.

In general, associated autoimmune diseases can be found in 13% of RDD. As demonstrated in the our case a subset of patients with RDD present increased levels of IgG4-bearing plasma cells upon immunohistological examination rendering the differential diagnosis with IgG4-related disease somewhat difficult [1, 14]. The overlap between RDD and IgG4-related disease with the two conditions sharing similar features, including emperipolesis, may represent a spectrum [15, 16], whereas other authors suggest differentiation between the two based on the degree of IgG4-positive infiltrates and IgG4:IgG ratio [17]. Menon et al. reported the presence of IgG4-bearing plasma cells in 28/70 RDD cases (40%) with 17.4% fulfilling the consensus guideline criteria for a histological diagnosis of IgG4-related disease [18]. Of note, the two cases of intrathoracic RDD with elevated IgG4-bearing plasma cells presented with pulmonary masses and not with cysts. The presence of abundant emperipolesis was highly suggestive for RDD and the levels of IgG4-bearing plasma cells insufficient to make a diagnosis of IgG4-related disease. However, the significance of IgG4-bearing plasma cells in RDD remains to be further elucidated, and their presence might indicate a better treatment response to corticosteroids and/or rituximab in case of disease progression.

In conclusion, this case report indicates a benign course of nodal RDD with thyroid and tracheal infiltration following surgical resection, describes an association of typical histological signs of RDD (emperipolesis) with IgG4-related disease features, and that lung cysts might be a manifestation of RDD.

## Abbreviations

BHDS: Birt-Hogg-Dube syndrome
LAM: lymphangioleiomyomatosis
LIP: lymphocytic interstitial pneumonia
PLCH: Pulmonary Langerhans cell histiocytosis
RDD: Rosai-Dorfman disease
VEGF-D: Vascular endothelial growth factor-D
